# Methylation and gene expression differences between reproductive and sterile bumblebee workers

**DOI:** 10.1002/evl3.129

**Published:** 2019-08-05

**Authors:** Hollie Marshall, Zoë N. Lonsdale, Eamonn B. Mallon

**Affiliations:** ^1^ Department of Genetics and Genome Biology The University of Leicester Leicester United Kingdom

**Keywords:** Bombus, bumblebee, expression, hymenoptera, methylation

## Abstract

Phenotypic plasticity is the production of multiple phenotypes from a single genome and is notably observed in social insects. Multiple epigenetic mechanisms have been associated with social insect plasticity, with DNA methylation being explored to the greatest extent. DNA methylation is thought to play a role in caste determination in *Apis mellifera*, and other social insects, but there is limited knowledge on its role in other bee species. In this study, we analyzed whole genome bisulfite sequencing and RNA‐seq data sets from head tissue of reproductive and sterile castes of the eusocial bumblebee *Bombus terrestris*. We found that genome‐wide methylation in *B. terrestris* is similar to other holometabolous insects and does not differ between reproductive castes. We did, however, find differentially methylated genes between castes, which are enriched for multiple biological processes including reproduction. However, we found no relationship between differential methylation and differential gene expression or differential exon usage between castes. Our results also indicate high intercolony variation in methylation. These findings suggest that methylation is associated with caste differences but may serve an alternate function, other than direct caste determination in this species. This study provides the first insights into the nature of a bumblebee caste‐specific methylome as well as its interaction with gene expression and caste‐specific alternative splicing, providing greater understanding of the role of methylation in phenotypic plasticity within social bee species. Future experimental work is needed to determine the function of methylation and other epigenetic mechanisms in insects.

**Impact Summary**

Social insects, such as ants, termites, bees, and wasps, can produce individuals with extreme physical and behavioral differences within the same colony known as castes (e.g., workers/soldiers/queens). These individuals have similar genomes and many studies have associated epigenetic mechanisms with the differences observed. Epigenetic modifications are changes that affect how genes are expressed without changing the underlying DNA code. Here, we investigated differences in DNA methylation (a well‐researched modified base) between different reproductive castes of the bumblebee, *Bombus terrestris*, an economically and environmentally important pollinator species. We found that *B. terrestris* has a similar methylation profile to other holometabolous insect species in terms of the distribution of methylation throughout the genome and the relationship between methylation and gene expression. Genes that have differences in methylation between reproductive castes are involved in multiple biological processes, including reproduction, suggesting methylation may hold multiple functions in this species. These differentially methylated genes are also different to differentially methylated genes identified between honeybee reproductive castes, again suggesting methylation may have a variable function. These findings provide a greater understanding of the role of methylation in caste determination in social insect species.

Phenotypic plasticity is the production of multiple phenotypes from a single genome. It plays a crucial role in the adaptive capabilities of species (Chevin et al. 2010) and is notably observed in social insects. Social insects exhibit sometimes extreme morphological and behavioral differences within a single colony, known as castes. The mechanisms by which species develop differences between castes are diverse; some species use only environmental cues, whereas others rely only on inherited changes, with many species falling somewhere in between these two extremes (Matsuura et al. 2018). For example, some ant species from the *Pogonomyrmex* genus have purely genetic caste determination (Mott et al. 2015). On the other hand, many ant species undergo caste determination in response to only the environment, indicating their genomes must contain the code for all caste possibilities, with the phenotype potentially determined by epigenetic factors (Bonasio et al. 2012).

Multiple epigenetic mechanisms have been associated with social insect plasticity. Histone modifications have been shown to be involved with plasticity, for example changes in histone acetylation alter the behavior of major workers of the ant species *Camponotus floridanus*, making them more similar to the behavior of minor workers (Simola et al. 2016). Variation in microRNA expression levels has been identified in both honeybee (Ashby et al. 2016) and bumblebee (Collins et al. 2017) castes. However, the most active research in this area has been focused on DNA methylation (Glastad et al. 2015).

DNA methylation is the addition of a methyl group to a cytosine nucleotide. In mammals, methylation primarily occurs in a CpG context (CpG referring to a cytosine base immediately followed by a guanine base); the percentage of CpGs methylated is usually over 70%, with methylation serving to repress gene expression when occurring in promoter regions (Feng et al. 2010). However, in insects, it is generally found in much lower quantities, ranging from zero methylation in most Diptera species studied to >2% in Hymenoptera and up to 14% in some species of Blattodea (Provataris et al. 2018). It is also enriched in gene bodies rather than throughout the genome, as in mammals (Fang et al. 2012; Wang et al. 2013), with a possible role in alternative splicing (Bonasio et al. 2012).

DNA methylation has been associated with the switching of worker castes in honeybees (Herb et al. 2012). A major finding was that silencing of the *Dnmt3* gene (involved in methylation establishment) in larvae produced queens rather than workers (Kucharski et al. 2008). DNA methylation has also been linked with alternative splicing differences between castes in two ant species (Bonasio et al. 2012) and is thought to be involved in caste determination in *Copidosoma koehleri*, a species of primitively social wasp (Shaham et al. 2016). However, it is clear DNA methylation is not a conserved mechanism in Hymenoptera for caste differentiation. No association between caste and methylation has been found in a number of wasp and ant species (Patalano et al. 2015; Standage et al. 2016). Additionally, the statistical methods of previous next generation sequencing analyses on social insect methylation have been brought into question (Libbrecht et al. 2016).

A greater variety of species are needed to begin to understand the role of DNA methylation in social insect caste determination. Here, we assess whole genome methylation differences between reproductive castes of the bumblebee, *Bombus terrestris*, with an aim to investigate the role of methylation in caste determination in this species. Bumblebees are primitively eusocial and are an important pollinator species, both economically and environmentally. They are generalist pollinators and are keystone species in some ecosystems (Woodard et al. 2015). *Bombus terrestris* colonies are annual and are founded by a singly mated queen in early spring; she will lay diploid eggs resulting in female workers and later switch to male haploid eggs, known as the switching point (Bloch 1999). A competition phase then occurs between queens and workers, where some workers will become reproductive and produce their own haploid sons (Alaux et al. 2006); this results in distinct reproductive worker castes within the colony. Multiple recent studies have highlighted *B. terrestris* as an ideal organism to assess methylation as a potential regulatory mechanism for reproductive caste determination (Amarasinghe et al. 2014; Lonsdale et al. 2017; Li et al. 2018).

Methylation regulatory genes were identified in the bumblebee genome and have since been shown to have varying expression levels between queens, workers and drones (Li et al. 2018). Additionally, genes showing allele‐specific methylation and gene expression have been identified and are enriched in reproductive‐related processes (Lonsdale et al. 2017). Finally, experimental changes in methylation in *B. terrestris* workers has been shown to alter levels of reproductive behavior (Amarasinghe et al. 2014). Although these studies highlight differences in methylation between *B. terrestris* castes, it is still unclear where those differences are within the genome and also whether methylation differences are related to changes in gene expression, potentially leading to caste differentiation.

In this study, we compared whole genome bisulfite sequencing (WGBS) data sets from reproductive and sterile worker castes of *B. terrestris*, allowing us to identify differences in methylation at base‐pair resolution throughout the genome. We then linked these data with gene expression data for the same individuals to identify a potential relationship between gene expression and methylation regarding reproductive caste determination. Within this study, we have also characterized the *B. terrestris* methylome to allow comparative analyses between castes. If methylation plays a role in caste determination, we would expect to find differentially methylated genes between castes, with functions related to reproduction. We would also expect any differentially methylated genes between castes to be enriched for differentially expressed genes or genes which have different exon usage between castes. Additionally, if there is a conserved role for methylation in caste determination in Hymenoptera, we would expect to find orthologous genes differentially methylated between *B. terrestris* reproductive castes and *Apis mellifera* reproductive castes.

## Methods

### BEE HUSBANDRY AND TISSUE SAMPLING

Three *B. terrestris* colonies, from Agralan, UK, were reared in constant red light at 26°C and 60% humidity. They were fed 50% v/v apiary solution (Meliose‐Roquette, France) and pollen (Percie du set, France) ad libitum. Callow workers, less than 24 hours old, were taken from each colony and placed in small rearing boxes of five individuals.

**Figure 1:**
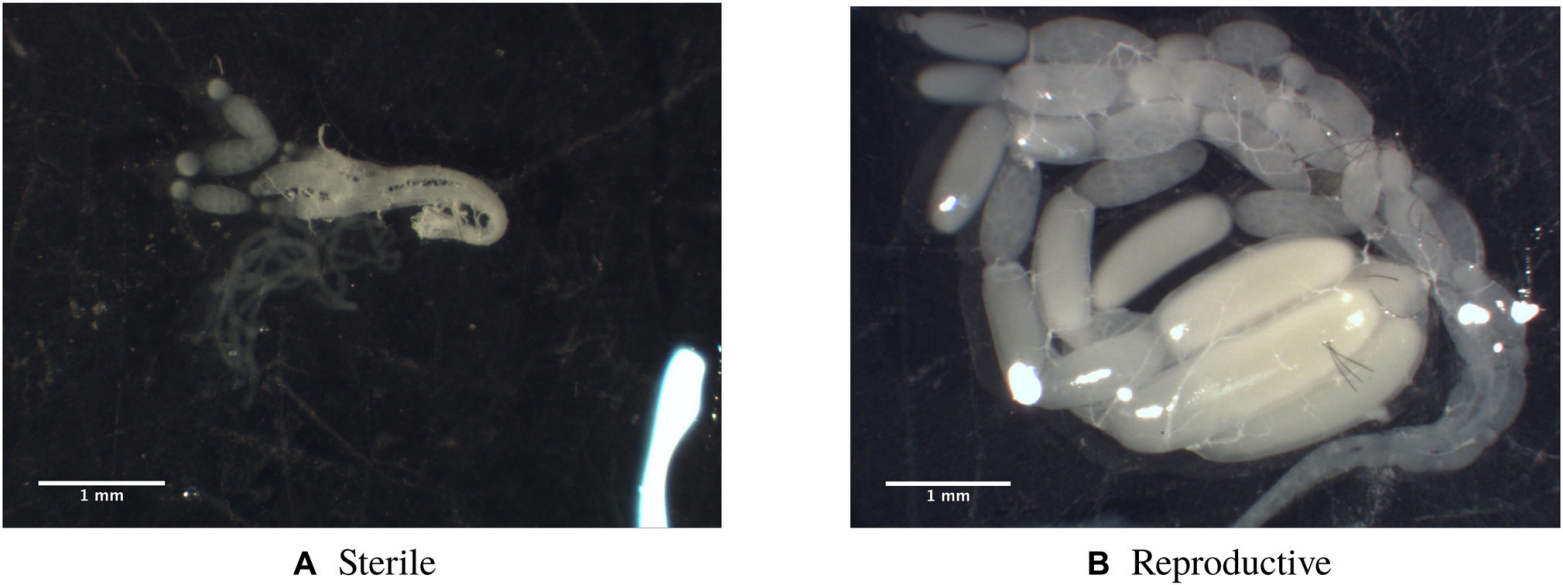
(A) one half of a pair of ovaries from a sterile bumblebee worker, with a score of 0. (B) One half of a pair of ovaries from a reproductive bumblebee worker, with a score of 4. Scores generated following Duchateau and Velthuis (1988).

The worker bees were sacrificed at six days old. For each bee, the head was snap frozen in liquid nitrogen. Through dissection in 1% PBS solution, the reproductive status of each bee was determined and classed as either reproductive, sterile, or intermediate. Workers were classed as having developed ovaries, and therefore reproductive, if the largest oocyte was larger than the trophocyte follicle (Duchateau and Velthuis 1988). This measurement is tightly correlated with reproductive status (Foster et al. 2004; Geva et al. 2005). The ovaries of each worker were weighed, and the length of the largest oocyte was measured using ImageJ version 1.50e (Schneider et al. 2012) (Supporting Information 1.0.0). Worker “reproductiveness” was classified on a scale from 0 to 4 based on Duchateau and Velthuis (1988), 0 begin completely sterile (Fig. 1A) and 4 having fully developed ovaries (Fig. 1B).

### RNA AND DNA EXTRACTION AND SEQUENCING

**Figure 2:**
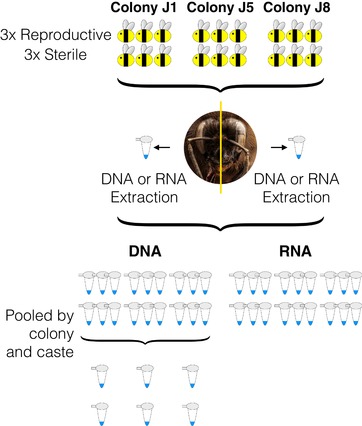
Overview of sample preparation for sequencing. Three reproductive workers and three sterile workers were selected from three colonies (J1, J5 and J8 represent colony names). Half of each head was randomly allocated for RNA/DNA extraction. All 18 RNA samples were sent for RNA‐Seq (three of each caste from each colony). DNA samples were pooled by colony and caste creating a representative reproductive and sterile sample per colony.

Three reproductive individuals and three sterile individuals from each of the three colonies were selected for RNA and DNA extraction (see Fig. 2 for an overview). Heads were cut in half (using a lateral incision central between the eyes). Each head half was randomly allocated for either DNA/RNA extraction to avoid left/right hemisphere bias. RNA was extracted using the Sigma‐Aldrich GenElute Mammalian Total RNA Miniprep kit and DNA was extracted using the Qiagen DNeasy blood and Tissue kit, individually for each half head per sample, following manufacturers protocols. The extracted RNA was treated with DNase and the extracted DNA was treated with RNase. DNA was pooled per colony and reproductive status, that is, the three reproductive samples from a single colony were pooled to create one representative reproductive sample for that colony (Fig. 2). RNA samples were processed individually. DNA and RNA quality and quantity were determined by Nanodrop and Qubit® fluorometers (Supporting Information 1.0.1 and 1.0.2). A total of 18 RNA samples (three individuals per reproductive status for each of the three colonies) were sent for 100 bp paired‐end sequencing and six pooled DNA samples (one sample per reproductive status per colony consisting of three individuals per pool) were sent for 100 bp paired‐end bisulfite sequencing on a HiSeq 2000 machine (Illumina, Inc.) by BGI Tech Solution Co., Ltd.(Hong Kong). Library preparation was carried out by BGI using their standard directional WGBS pipeline. A 1% lambda spike was included as an unmethylated control in each WGBS library.

### DIFFERENTIAL EXPRESSION AND ALTERNATIVE SPLICING

Low‐quality bases were removed from the RNA‐Seq libraries using CutAdapt version 1.1 (Martin 2011). Reads were aligned to the reference genome (Bter_1.0, Refseq accession no. GCF_000214255.1; Sadd et al. 2015) using STAR version 2.5.2 (Dobin et al. 2016) with standard parameters. Reads were counted per gene using HTseq version 0.10.0 (Anders et al. 2015). A differential expression analysis was then carried out after count normalization via a negative binomial generalized linear model (GLM) implemented by DEseq2 version 1.20.0 (Love et al. 2014) in R version 3.4.0 (http://www.R‐project.org) with colony and reproductive status as independent variables. Genes were classed as differentially expressed when *q* < 0.05 after correction for multiple testing using the Benjamini–Hochberg method (Benjamini and Hochberg 1995).

Differential exon expression was determined using the DEXseq version 1.26.0 R package (Anders et al. 2012); briefly this package calculated the ratio of expression of a given exon in a given gene relative to other exons in the same gene for each sample. The relative exon expression per colony was then calculated taking into account dispersion between colonies. A GLM was then used to test for a difference in the relative proportion of expression of each exon between castes, accounting for sample differences and overall gene expression differences between castes; *P*‐values were corrected for multiple testing using the Benjimini–Hochberg method (Benjamini and Hochberg 1995) and exons were classed as differentially used between castes when *q* < 0.1. The benefit of this method over general alternative splicing analysis is that specific differentially used exons can be identified between castes allowing the relationship with exonic methylation to be investigated.

### DIFFERENTIAL METHYLATION

BS‐seq libraries were aligned to the reference genome (Bter_1.0, Refseq accession no. GCF_000214255.1; Sadd et al. 2015) using Bismark version 0.16.1 (Krueger and Andrews 2011) and bowtie2 version 2.2.6 (Langmead and Salzberg 2012) with standard parameters. Bismark was also used to extract methylation counts and carry out deduplication. Annotation of the methylation counts with genomic features (from the *B. terrestis* annotation file, Refseq accession no. GCF_000214255.1) was carried out using custom R scripts implementing the sqldf version 0.4.11 library (Grothendieck 2017). Bisulfite conversion efficiency was calculated by aligning reads to the lambda reference genome (Refseq accession no. GCF_000840245.1) and calculating the single‐site methylation level, as in Schultz et al. (2012).

Prior to differential methylation analysis, coverage outliers (above the 99.9th percentile) were removed along with bases covered by less than 10 reads. The methylation status of each CpG was then determined using the “methylation status calling” (MSC) procedure, as described in Cheng and Zhu (2014). Briefly, this involves applying a mixed binomial model to each CpG, which includes estimation of both the false discovery rate (FDR) and the non‐false discovery rate to make a binary methylation call per site. CpG sites were then filtered to remove any site that did not return as methylated in at least one sample. This functions to reduce the number of tests and hence decreases the required stringency of the later FDR correction applied during differential methylation testing. This is a vital step for species, such as bumblebees, with extremely low genome methylation where the majority of sites show zero methylation in all samples. A logistic regression model was then applied via the R package methylKit version 1.6.1 (Akalin et al. 2012) to determine differentially methylated sites, taking into account colony as a covariate due to high intercolony variation (see Supporting Information 2.0). A minimum difference of at least 10% methylation and a *q*‐value of <0.05 were required for a single site to be classed as differentially methylated. Genes containing at least one differentially methylated CpG and a minimum weighted methylation difference (Schultz et al. 2012) of 10% across the entire gene were classed as differentially methylated between reproductive castes.

We chose not to include a permutation test as part of the differential methylation analysis, as has been seen in previous research (Libbrecht et al. 2016; Arsenault et al. 2018), although it is included in our Supporting Information data. There is structure present in our data due to high methylation variation between colony replicates. When structure is present within data, permutation tests do not produce reliable outcomes, as discussed in Winkler et al. (2015). A higher number of replicates would allow label shuffling within confounding factors, maintaining the structure of the data, thus allowing a valid permutation test (see Supporting Information 2.0).

### GENE ONTOLOGY ANALYSIS

Gene ontology (GO) terms for *B. terrestris* were taken from a custom database made in Bebane et al. (2019). GO enrichment analysis was carried out using a hypergeometric test with Benjamini–Hochberg multiple‐testing correction (Benjamini and Hochberg 1995). GO terms were defined as enriched when *q* < 0.05. GO terms from differentially methylated genes were tested for enrichment against GO terms associated with all methylated genes. Genes were classed as methylated when the weighted methylation score per gene was greater than zero (Schultz et al. 2012). Additionally, the GO terms associated with hypermethylated genes in either sterile or reproductive workers were tested for enrichment against the GO terms associated with all differentially methylated genes between castes to determine if there are different functions for hypermethylated genes in either sterile or reproductive workers. GO terms for differentially expressed genes and genes containing different exon usage between castes were tested for enrichment against GO terms associated with all genes identified in the RNA‐seq data. REVIGO (Supek et al. 2011) was used to obtain the GO descriptions from the GO identification numbers.

### COMPARATIVE ANALYSES

The hypergeometric test was applied to gene lists from the various analyses to determine if any overlaps were statistically significant. Custom R scripts were used to investigate the relationship between gene expression and methylation. A reciprocal blast between the honeybee (Amel_4.5, Refseq accession no. GCA_000002195.1) and bumblebee genome (Bter_1.0, Refseq accession no. GCA_000214255.1) was carried out using blast+ version 2.5.0 (Camacho et al. 2009), where the fasta sequence for each gene of each species was blasted against a custom database containing the fasta sequence for every gene of the opposite species, allowing only one match per gene and a minimum *e*‐value of 1 × 10^−3^. Gene matches were then filtered to ensure only matches that occurred in both directions and to the same gene were kept. For example, multiple honeybee genes matched the same bumblebee gene, therefore all of these matches were discarded. This allowed us to construct a database of putative orthologous genes. A custom script was then used to check for overlap between the differentially methylated genes identified here and differentially methylated genes identified in Lyko et al. (2010) between honeybee reproductive castes.

## Results

### GENOME‐WIDE METHYLATION DIFFERENCES BETWEEN CASTES

Up to a maximum of 10 bp were trimmed from the start of all reads due to base bias generated by the Illumina sequencing protocol (Krueger et al. 2011). The mean mapping efficiency was 63.6 ± 1.4% (mean ± SD) and the mean coverage was 17.7 ± 0.5 reads per base; the average number of uniquely mapped reads was 27,709,214 ± 753,203. The mean bisulfite conversion efficiency, calculated from the unmethylated lambda spike, was 99.55 ± 0.02%. After accounting for the conversion efficiency, there were no methylated cytosines in a non‐CpG context. The mean single site methylation level (Schultz et al. 2012) in a CpG context was determined as 0.22 ± 0.07%, calculated from the number of methylated cytosines divided by the sum of methylated and unmethylated cytosines and accounting for bisulfite conversion efficiency.

**Figure 3:**
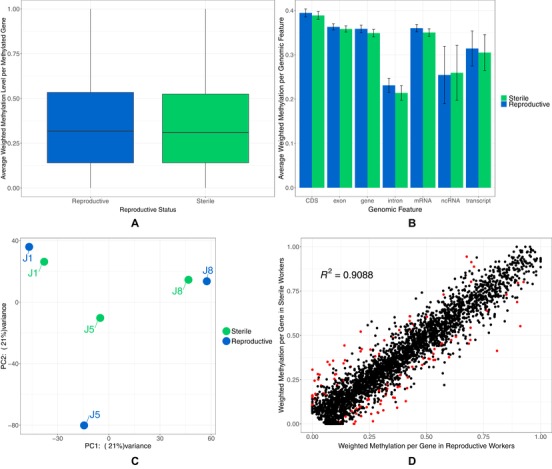
(A) Box plot of the mean weighted methylation level of methylated genes (*n* = 3412) across colonies for each caste. (B) The mean weighted methylation level across colonies for each genomic feature for both reproductive and sterile workers. Error bars are 95% confidence intervals of the mean. (C) PCA plot generated by methylKit showing samples cluster by colony using per site CpG methylation. (D) Scatter plot of the average weighted methylation level (across colonies) for reproductive workers against sterile workers. Each dot represents a gene and each red dot represents a differentially methylated gene (*q* < 0.05 and a minimum gene weighted methylation difference of 10%).

A total of 3412 genes were classed as methylated, that is, they had a weighted methylation level >0 in at least one sample. There was no significant difference in the overall weighted methylation level of the methylated genes between reproductive and sterile workers (Mann–Whitney *U* test: *W* = 5,948,300, *P* = 0.1172, Fig. 3A). GO terms enriched in methylated genes compared to all genes annotated in the genome (*q* < 0.05) include a large variety of biological processes (Supporitng Information 1.0.5). Specifically, *posttranscriptional regulation of gene expression* (GO:0010608) and *histone modification* (GO:0016570) are enriched as well as terms related to reproductive processes, for example, *reproduction* (GO:0000003) and *oogenesis* (GO:0048477).

There was no significant difference in the weighted methylation level of all genomic features between reproductive and sterile workers (two‐way analysis of variance [ANOVA], interaction between genomic feature and reproductive status; *F*_1, 6_ = 0.637, *P* = 0.701, Fig. 3B), we also tested for differences in methylation levels of putative promotors but as promotor regions are currently unannotated for the current genomic annotation we feel these results are not reliable (Supporting Information 2.1, Fig. S3a). Irrespective of worker reproductive status, we found methylation differences between genomic features (Kruskal–Wallis; chi‐squared = 729.35, df = 7, *P* < 2.2 × 10^−16^), with methylation being significantly enriched in coding regions compared to introns and ncRNAs (Supporting Information 1.0.6).

We also found no difference in the weighted methylation level across the genome per linkage group between reproductive and sterile workers (two‐way ANOVA, interaction between linkage group and reproductive status; *F*_1, 17_ = 0.034, *P* = 1.0, Supporting Information 2.1, Fig. S3b). Weighted methylation did vary significantly between linkage groups within the genome irrespective of reproductive status (Kruskal–Wallis chi‐squared = 131.59, df = 17, *P* < 2.2 × 10^−16^, Supporting Information 1.0.6), however due to the number of unplaced scaffolds these results should be interpreted with care.

Finally, using CpG methylation levels, samples cluster by colony rather than by reproductive caste (Fig. 3C). This indicates high intercolony variation in methylation.

### GENE LEVEL METHYLATION DIFFERENCES BETWEEN CASTES

A total of 4,681,131 CpG sites had a coverage >10 in all six sample data sets, of those 16,194 returned as methylated in at least one sample after running the MSC procedure. A total of 624 of these CpGs were identified as differentially methylated between reproductive castes, 613 of these were located in a total of 478 genes (Supporting Information 1.0.4). Note that 11 differentially methylated CpGs were located outside of genes, nine of those were within 5000 bp upstream or downstream of a gene with no apparent trend in the expression of near‐by genes (Supporting Information 1.0.5). 111 genes contained a differentially methylated CpG and also had a weighted methylation difference of 10% between reproductive and sterile workers (Supporting Information 1.0.7, Fig. 3D).

Of the 111 differentially methylated genes, there was no preference for genes to be hypermethylated in either reproductive or sterile workers (chi‐squared goodness of fit, X‐squared = 2.027, df = 1, *P* = 0.1545), with 63 genes hypermethylated in reproductive workers and 48 genes hypermethylated in sterile workers.

GO terms enriched in differentially methylated genes compared to all methylated genes (*q* < 0.05) contained a variety of biological processes (Supporting Information 1.0.8), among these processes were terms involved with reproduction, including *meiotic cell cycle* (GO:0051321) and *female germline ring canal stabilization* (GO:0008335). One of the genes associated with the above GO terms is *eggless* (LOC100647514), which shows hypermethylation in sterile workers. This gene contains a Methyl‐CpG binding domain, which has been associated with histone H3, lysine 9‐specific methyltransferase that contributes to repression of transcription (Wakefield et al. 1999).

There were no specific GO terms enriched for either the hypermethylated genes in sterile workers or the hypermethylated genes in reproductive workers compared to all differentially methylated genes as a background.

### EXPRESSION DIFFERENCES BETWEEN CASTES

All reads had 13 bp trimmed from the start due to base bias generated by the Illumina protocol (Krueger et al. 2011). The mean percentage of uniquely mapped reads was 89.4 ± 0.8% (mean ± SD). This equated to a mean of 10,115,366 ± 1,849,600 uniquely mapped reads. After running a differential expression analysis with DESeq2, the decision was made to remove one sample from all downstream analysis due to possible mislabeling of reproductive status (Supporting Information 2.2).

**Figure 4:**
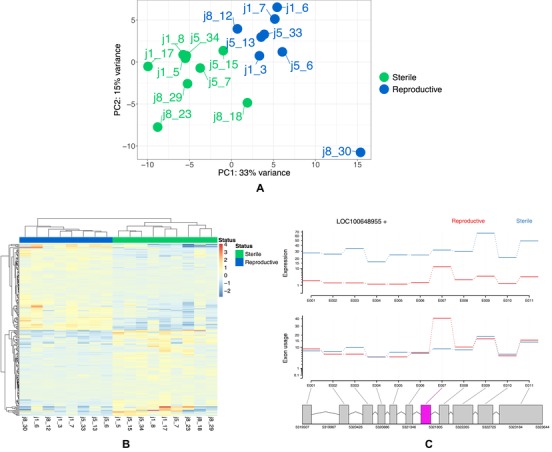
(A) PCA plot showing samples cluster by caste for gene expression, the first half of each label represents the colony name and the second half is the individual identification number. (B) Heatmap showing the 100 top differentially expressed genes between reproductive castes, samples cluster by reproductive status. Sample names are shown at the bottom of the plot. (C) An example of a gene that shows differential exon expression in one exon between reproductive castes. The top section of the plot shows the general expression differences between castes, the second section shows the normalized counts per exon (given expression differences) and the third section highlights the differentially expressed exon in pink. Gene shown: *probable peroxisomal acyl‐coenzyme A oxidase 1* (ID: LOC100648955).

Samples cluster by reproductive status when the expression of all genes is assessed (Fig. 4A). A total of 334 genes were identified as differentially expressed (*q* < 0.05, Fig. 4B). There was no difference in the number of upregulated genes in either reproductive or sterile workers (chi‐squared goodness of fit: X‐squared = 0.2994, df = 1, *P* = 0.5843), with 172 genes upregulated in reproductive workers and 162 genes upregulated in sterile workers.

One of the most upregulated genes in reproductive workers was vitellogenin (gene ID: LOC100650436, log2 fold‐change of 2.92, *q* = 4.85 × 10^−6^). Previous work has found upregulation of this gene in reproductive *B. terrestris* workers is linked to aggressive behavior rather than directly to ovary development (Amsalem et al. 2014). Additionally, two genes coding for serine‐protease inhibitors were found to be upregulated in reproductive workers; these proteins have been linked to reproduction in other insect species (Bao et al. 2014).

Enriched GO terms associated with the differentially expressed genes compared to the background of all genes in the RNA‐seq data (*q* < 0.05) contained a variety of biological processes, including reproductive‐related terms (Supporting Information 1.1.0). Additionally, there were no specific GO terms enriched in upregulated genes of reproductive workers compared to all differentially expressed genes as the background. However, there were two GO terms enriched for upregulated genes in sterile workers compared to differentially expressed genes as the background, these were as follows: *cellular lipid metabolic process* (GO:0044255) and *isoprenoid biosynthetic process* (GO:0008299).

A total of 59 genes were identified as having differential exon usage, containing 83 differentially expressed exons between reproductive castes (*q* < 0.1, Supporting Information 1.1.1, see example Fig. 4C). There is no difference in the number of upregulated exons in reproductive workers compared to sterile workers (chi‐squared goodness of fit: X‐squared = 3.4819, df = 1, *P* = 0.06204), with reproductive workers having 33 upregulated exons and sterile workers having 50 upregulated exons. The enriched GO terms associated with genes containing differentially used exons compared to the background of all genes in the RNA‐seq data (*q* < 0.05) contained a variety of biological processes (Supporting Information 1.1.2), however there were no GO terms with a clear connection to reproductive processes.

### RELATIONSHIP OF METHYLATION AND GENE EXPRESSION

**Figure 5:**
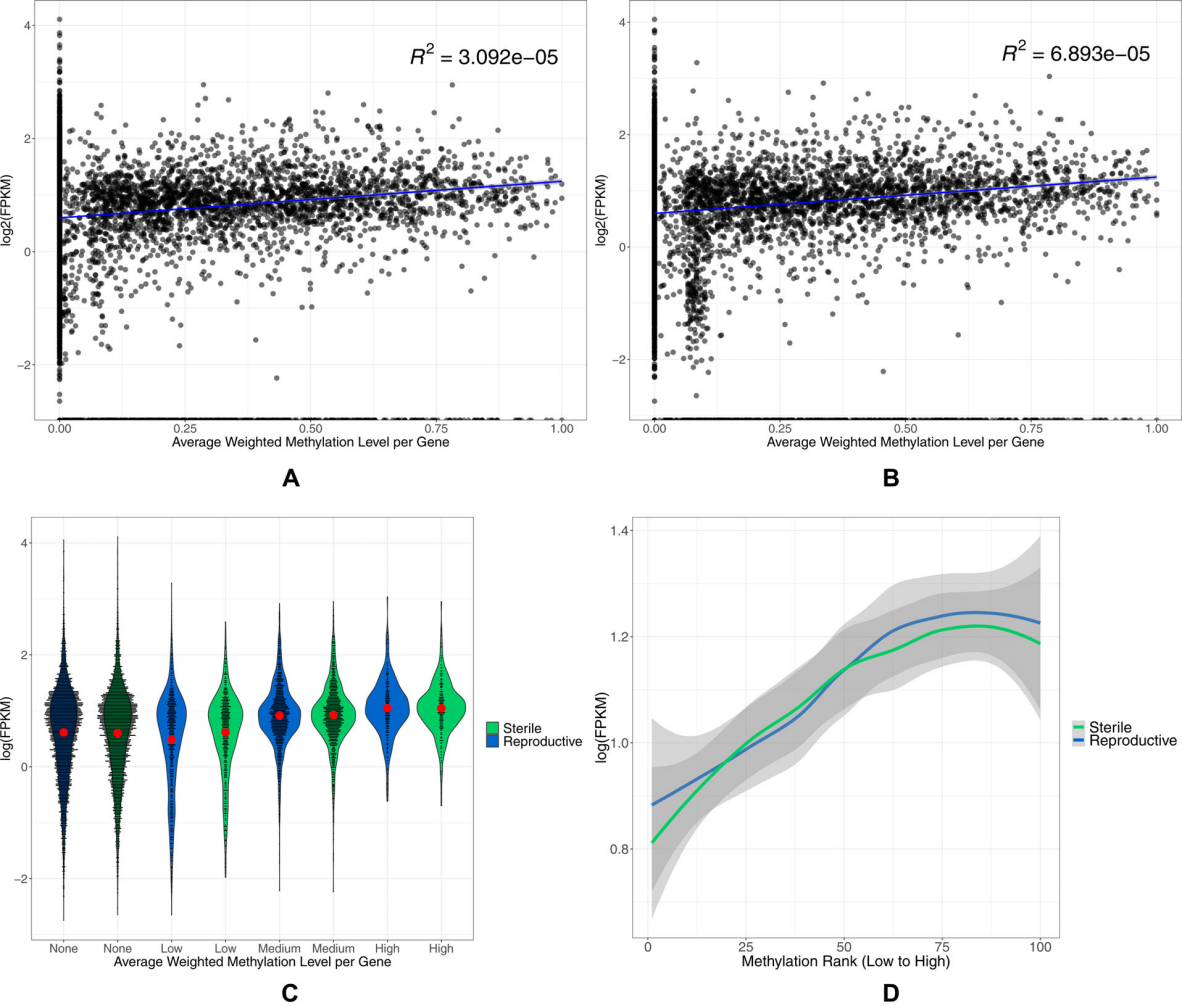
(A and B) The colony average weighted methylation level for every gene plotted against the log(FPKM) of that gene for sterile workers and reproductive workers, respectively. Each black dot represents a single gene. The blue line is a fitted linear regression with the gray shaded area representing 95% confidence intervals. (C) Violin plots showing the distribution of the data via a mirrored density plot, meaning the widest part of the plots represent the most genes. Weighted methylation level per gene per caste, averaged across colonies, was binned into four categories, no methylation, low (>0–0.2), medium (0.2–0.7), and high (0.7–1), as in Liu et al. (2019). The red dot indicates the mean with 95% confidence intervals. Each black dot represents a single gene. (D) Binned methylated genes (*n* = 3412, 100 being the most highly methylated) based on the mean weighted methylation level across colonies per reproductive caste, plotted against the log(FPKM) expression level per gene. Data were smoothed using the LOESS method, and gray areas are 95% confidence intervals.

On an individual gene basis, methylation and reproductive caste have no effect on expression level (Fig. 5A and 5B, linear mixed effects model with colony as a random factor; methylation: df = 49172, *t* = −1.295, *P* = 0.195, reproductive status: df = 49172, *t* = −0.638, *P* = 0.524, interaction between methylation and reproductive status: df = 49172, *t* = 0.112, *P* = 0.911).

However, gene groups with varying methylation levels show different levels of expression (Kruskal–Wallis; chi‐squared = 131.59, df = 17, *P* < 2.2 × 10^−16^; Fig. 5C). Specifically genes with no methylation show higher expression than genes classed as lowly methylated but lower expression than genes classed as medium/high in terms of methylation (Dunn's test with Benjamini–Hochberg multiple‐testing correction; no methylation vs. low methylation: *Z* = −13.14, *P* = 4.09 × 10^−39^, no methylation vs. medium methylation: *Z* = 4.5, *P* = 6.82 × 10^−6^, no methylation vs. high methylation: *Z* = 7.32, *P* = 3.86 × 10^−13^; Fig. 5C). Reproductive caste still has no effect on gene expression in relation to methylation status when genes are grouped (two‐way ANOVA, interaction between reproductive status and methylation level; *F*_1, 3_ = 0.017, *P* = 0.99).

A linear mixed effects model was then applied to assess the relationship between gene expression, methylation, and reproductive status for only methylated genes using colony as a random factor. There is a positive relationship between gene expression and methylation in methylated genes with reproductive status having no effect (Fig. 5D, methylation: df = 17,390, *t* = 6.154, *P* = 7.72 × 10^−10^, reproductive status: df = 17,390, *t* = −0.328, *P* = 0.743, interaction between methylation and reproductive status: df = 17,390, *t* = −0.200, *P* = 0.842).

### RELATIONSHIP OF METHYLATION AND DIFFERENTIAL GENE EXPRESSION

**Figure 6:**
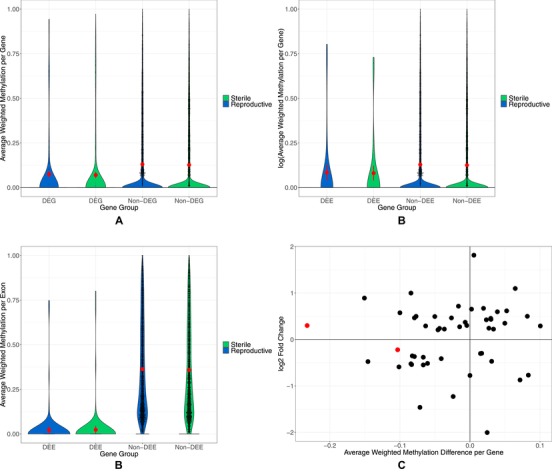
(A) Violin plots showing the distribution of the data via a mirrored density plot, meaning the widest part of the plots represent the most genes. The red dots represent the mean of each gene set along with error bars representing 95% confidence intervals of the mean. Each black dot is an individual gene. The mean weighted methylation per gene across colonies per caste is plotted for either differentially expressed genes (DEG) or nondifferentially expressed genes (non‐DEG). (B) Violin plots of the mean weighted methylation per gene across colonies per caste is plotted for either genes containing differentially expressed exons (DEE) or genes with non‐differentially expressed exons (non‐DEE). (C) Violin plots of the mean weighted methylation per exon across colonies per caste for DEE and non‐DEE. The red dots represent the mean of each exon set along with error bars representing 95% confidence intervals of the mean. Each black dot is an individual exon. (D) Scatter plot of the difference in the mean weighted methylation level across colonies between castes plotted against the log2 fold change in expression of differentially expressed genes between castes. Each dot represents a gene; only genes which have a methylation difference >0 are shown. The red dots indicate that the gene is also differentially methylated.

Weighted methylation differences between differentially expressed genes and nondifferentially expressed genes were assessed along with weighted methylation differences between genes containing differentially expressed exons and genes without differentially expressed exons (Fig. 6A and 6B). Differentially expressed genes and genes containing differentially expressed exons between castes show lower methylation than nondifferentially expressed genes or genes containing no differentially expressed exons, with reproductive status and the interaction of reproductive status with gene expression type having no effect (Table 1, Fig. 6A and 6B). When weighted methylation is assessed per exon, differentially expressed exons have lower weighted methylation than nondifferentially expressed exons (Fig. 6C), with reproductive status and the interaction of reproductive status and exon expression having no effect (Table 1).

**Table 1:** Summary statistics of the linear models used to check for differences in weighted methylation level between gene sets by taking into account reproductive status

	Res. Df	RSS	Df	Sum of square	F	*P*
**Differentially expressed genes**						
Interaction vs. main effects model	16487	833.1	−1	−2.98 × 10^−4^	5.9 × 10^−3^	0.93
DEG vs. non‐DEG	N/A	835.14	1	2.04	40.38	2.147 × 10^−10^ *
Reproductive vs. sterile	N/A	833.15	1	0.049	0.97	0.32
**Genes with differentially expressed exons**						
Interaction vs. main effects model	16409	832.69	−1	−7.93 × 10^−6^	2.00 × 10^−4^	0.99
DEE vs. non‐DEE	N/A	832.92	1	0.23	4.59	0.032*
Reproductive vs. sterile	N/A	832.74	1	0.048	0.95	0.33
**Differentially expressed exons**						
Interaction vs. main effects model	11301	780.36	−1	−1.39 × 10^−3^	0.02	0.89
DEE vs. non‐DEE	N/A	799.69	1	19.33	279.97	<2.00 × 10^−16^ *
Reproductive vs. sterile	N/A	780.42	1	0.06	0.86	0.35

*A significant *P*‐value <0.05. DEG, differentially expressed genes; DEE, differentially expressed exons. The interaction versus main effects models were tested using the *anova* function in R to assess the interaction effect between gene set and reproductive status.

Of the 334 differentially expressed genes, 50 also showed some level of weighted methylation difference between reproductive and sterile workers (weighted methylation difference >0) (Fig. 6D). However, there is no relationship between the level of differential methylation and the level of differential expression for these 50 genes (linear model: *F*_1, 58_ = 0.2717, *P* = 0.6046).

**Figure 7:**
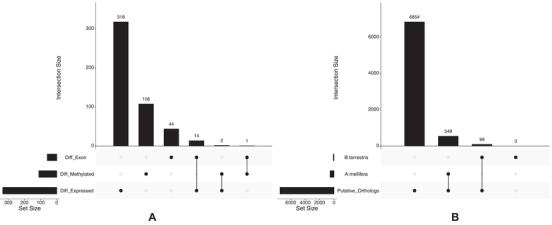
(A) UpSet plot showing the number of common genes between analyses. The set size indicates the number of genes in each category; differentially expressed, differentially methylated or genes containing a differentially expressed exon. The intersection size indicates the number of genes either unique to each set or the number common between sets. A single dot in the lower panel indicates the number of genes unique to the corresponding set and joining dots indicate the number of genes in common between the corresponding sets. (B) UpSet plot showing the number of putative orthologs between *A. mellifera* and *B. terrestris* along with the number of differentially methylated genes identified in Lyko et al. (2010) and in this study which are present in the putative ortholog database.

Gene lists were checked for potential overlap from all analyses. There was no significant overlap between differentially methylated genes and differentially expressed genes (two genes, hypergeometric test; *P* = 0.658, Fig. 7A). There was also no significant overlap between differentially methylated genes and genes containing differentially expressed exons (one gene, hypergeometric test; *P* = 0.12; Fig. 7A).

There was a significant overlap of genes found to be differentially expressed with those containing differentially expressed exons, 14 total (hypergeometric test; *P* = 1.44 × 10^−10^; Fig. 7A). All lists of overlapping genes can be found in Supporting Information 1.1.3.

### HONEYBEE ORTHOLOGOUS DIFFERENTIALLY METHYLATED GENES

Custom honeybee and bumblebee putative ortholog databases were created from 15,314 and 10,339 annotated genes, respectively (Amel_4.5 GCA_000002195.1, Bter_1.0 GCA_000214255.1). Note that 9,244 honeybee genes matched at least one bumblebee gene and 7,985 bumblebee genes matched at least one honeybee gene with an *e*‐value of <1 × 10^−3^. A total of 7,345 genes made the same match in both blast searches. Of these genes, 392 matched more than one gene in one or both blasts and were therefore removed. This left a final putative ortholog list of 6,953 genes. A total of 99 of the 111 differentially methylated genes identified here were present in the final putative ortholog list, however none of them matched the 549 genes identified as differentially methylated between honeybee reproductive castes by Lyko et al. (2010) (Fig. 7B).

## Discussion

We have used whole genome bisulfite sequencing and gene expression libraries from the same individual *B. terrestris* workers to investigate the role of methylation in caste determination. We found both reproductive and sterile workers show similar methylation patterns to other holometabolous insects. Methylation also has a similar relationship with gene expression compared to most other holometabolous insects currently studied, with more highly methylated genes showing higher levels of expression and lower levels of methylation being associated with differentially expressed genes. We found no methylation differences on a genomic scale between castes, however 111 genes were differentially methylated. These were involved in a variety of functions including reproductive related processes. We found no relationship between genes that are differentially expressed, or contain differential exon usage between castes with those that show differential methylation between castes. Finally, we also found no common putative orthologous genes differentially methylated between *B. terrestris* and *A. mellifera* reproductive castes.

This is the first data set to accurately quantify methylation at base‐pair resolution for *B. terrestris* castes. It confirms low methylation levels throughout the genome as predicted by Sadd et al. (2015). These low levels along with enrichment for CpG methylation in coding regions are also seen in many social insect species, including *A. mellifera* (Lyko et al. 2010) and multiple ant species (Bonasio et al. 2012; Libbrecht et al. 2016). These trends are also seen more generally in holometabolous insects (Provataris et al. 2018). However, they are not completely conserved among all holometabolous insects, for example the primitively social wasp species *Polistes dominula* shows 6% CpG methylation (Weiner et al. 2013), and the highly social termite, *Zootermopsis nevadensis*, has exceptionally high methylation levels compared to the majority of insects (Bewick et al. 2017), with 12% CpG methylation, and methylation being just as common in introns as exons (Glastad et al. 2016).

Higher levels of CpG methylation are associated with higher levels of gene expression in both *B. terrestris* reproductive castes. This is also the case in some other social insects, with Figure 5A and 5D showing almost identical trends to those found in Bonasio et al. (2012), Patalano et al. (2015), and Libbrecht et al. (2016). Additionally, other social insect species show higher methylation in nondifferentially expressed genes as we found here, for example *Dinoponera quadriceps* (Patalano et al. 2015), *Polistes canadensis* (Patalano et al. 2015), *Zootermopsis nevadensis* (Glastad et al. 2016), and *Cerapachys biroi* (Libbrecht et al. 2016). Higher levels of methylation in more highly expressed genes and in nondifferentially expressed genes is thought to indicate a role for methylation in housekeeping genes in some holometabolous insects (Foret et al. 2009; Lyko et al. 2010; Bonasio et al. 2012; Wang et al. 2013; Provataris et al. 2018). High levels of gene body methylation is also found in highly expressed genes in plants; while currently the function is unknown, Zilberman (2017) hypothesizes that it functions to stabilize expression by reducing histone variants and Bewick and Schmitz (2017) hypothesize that it is a by‐product of transposable element silencing.

We predicted that if methylation plays a role in reproductive caste determination, we would find differentially methylated genes between castes with reproductive related functions. We found 111 differentially methylated genes that include enriched GO terms for various reproductive‐related processes; this suggests methylation has some association with the switch between sterility and reproduction in *B. terrestris*. This supports previous work that exposed *B. terrestris* to a chemical that decreases general methylation levels and found workers were more likely to become reproductive (Amarasinghe et al. 2014), however we did not find a difference in the genome‐wide methylation levels of sterile and reproductive workers. It is also worth noting a worker classed as reproductive appeared to show a sterile transcriptional profile and this was included in the pool for the reproductive sample for colony J8. This will have “diluted” the strength of the methylation profile for this particular sample. It is therefore likely our data contain false negatives, meaning there may be differentially methylated genes between reproductive castes that do not appear in our data set.

Although we found differentially methylated genes between castes, we found no evidence for a relationship between methylation and gene expression in relation to reproductive and sterile workers. Only a small nonsignificant number of genes are both differentially methylated and differentially expressed between castes and there is no relationship between the degree of differential methylation and differential expression on a gene level. Previous research using the milkweed bug (*Oncopeltus fasciatus*) found knocking down *Dnmt1*, the gene responsible for DNA methylation maintenance, in ovary tissue with RNAi had no effect on gene expression, however these individuals could no longer reproduce (Bewick et al. 2019). This suggests methylation may play an alternative role, rather than direct regulation of gene expression, in reproduction of some insects. Bewick et al. (2019) suggest this role may be the regulation of genome stability and/or the regulation of vital cellular processes. The variety of GO terms involved in biological processes we obtained for the differentially methylated genes between castes supports this idea.

Additionally, we observed high intercolony variation in methylation, however we did not have sufficient replicates to test for exact differences between colonies. High intercolony variation could suggest that methylation may also play a role in the adaptive abilities of *B. terrestris*. Stable environmentally induced “epialleles” have been proposed to act as an additional layer of information for which selection can act upon (Flores et al. 2013). However, we currently do not know if *B. terrestris* methylation shows transgenerational inheritance or whether a large proportion is wiped during development, as in mammals (Messerschmidt et al. 2014).

It has also been suggested methylation may regulate alternative splicing, rather than expression in some insects (Glastad et al. 2011). We found no evidence here for the role of exon methylation in exonic expression differences between castes. Previous research using honeybees however did find an association between methylation and caste specific alternative splicing. Methylation differences between queens and workers in *A. mellifera* have been associated with caste‐specific splicing events (Lyko et al. 2010). Additionally, a knock‐down of *Dnmt3* by RNA interference was found to affect alternative splicing patterns in *A. mellifera*, with decreased methylation levels being directly related to exon skipping and intron retention (Li‐Byarlay et al. 2013). However, another social insect species, the primitively social wasp, *Polistes dominula*, has also shown no direct association between methylation and alternative splicing (Standage et al. 2016), however this species shows extremely low genome methylation and appears to lack the *Dnmt3* gene responsible for de novo methylation, suggesting that the link between methylation and alternative splicing in insects is variable.

Exon methylation has been shown to play a role in histone modifications and nucleosome stability in mammals (Jones 2012; Singer et al. 2015). These modifications have the ability to affect alternative splicing patterns through RNA polymerase accessibility, meaning while changes in DNA methylation may not be observed as directly related to alternative splicing, it is possible these changes have a downstream effect leading to transcriptional changes (Hunt et al. 2013). The analysis of the relationship between methylation and alternative splicing done here could be elaborated on further to include noncaste specific splicing sites and to also potentially identify the role of exon methylation in other epigenetic processes, which may themselves lead to alternative splicing.

It is also worth noting other epigenetic mechanisms may play a role in caste determination, for example microRNAs have been associated with caste switching in *A. mellifera* (Ashby et al. 2016). Additionally, Simola et al. (2016) found histone acetylation differences between worker castes of *Camponotus floridanus*. They inhibited histone acetylation and found this caused the major worker caste to behave more like a minor worker. This same species has also been shown to have caste specific methylation profiles (Bonasio et al. 2012). These examples indicate that it is likely an interplay between multiple mechanisms that ultimately cause social insect caste differentiation, again supported by the fact we find no association between methylation and caste‐specific alternative splicing.

Our final prediction was that differentially methylated genes between worker *B. terrestris* castes would be similar to those found to be differentially methylated between *A. mellifera* reproductive castes if methylation was involved in caste determination in Hymenoptera; we did not find any putative orthologous in common. This supports the idea in Bewick et al. (2019) that methylation may not directly influence caste determination. However, the differentially methylated gene list obtained for *A. mellifera* used queen samples to represent the reproductive caste (Lyko et al. 2010), whereas here reproductive worker samples were used; this could also explain the lack of agreement.

Considerably more experimental research is needed to better define the relationship between epigenetic processes and caste determination in social insects. Future work should focus on the consequences of experimental methylation removal or addition (Pegoraro et al. 2017), as well as exploring additional epigenetic mechanisms to attempt to identify a full pathway leading to reproductive caste differences. For example, CRISPR has recently been used to knockout two sex‐determining genes in *A. mellifera* causing individuals to change gender (Mcafee et al. 2019). This technology has also been adapted to be able to change the methylation state of a given loci (Vojta et al. 2016), allowing the possibility of exploring the function of methylation in specific genes.

Overall, we have found that the *B. terrestris* methylome appears similar to some other holometabolous insects in terms of overall levels and the relationship with gene expression. We found no genome‐wide methylation differences between reproductive castes, however we did find differentially methylated genes between reproductive castes, with GO terms enriched in many biological processes including reproduction. These results combined with previous research (Amarasinghe et al. 2014) indicate an association between methylation and reproductive caste differences in *B. terrestris*. However, it is clear, owing to the lack of consistency between differentially methylated genes and differentially expressed genes, methylation is not directly responsible for the associated changes in gene expression leading to the different reproductive phenotypes in *B. terrestris*. Additionally, the lack of similarity between differentially methylated genes between castes in *B. terrestris* and between castes in *A. mellifera* suggests that methylation may not directly contribute to caste determination in some Hymenoptera. Future work should focus on the experimental manipulation of epigenetic processes, such as methylation, in social insects to clarify functional roles within and across species.

Associate Editor: Z. Gompert

## Supporting information


**Supporting Information**

**Figure S1**. Histogram of the number of differentially methylated sites obtained from 10,000 permutations.
**Figure S2**. (a) PCA plot showing samples cluster more closely by colony than by reproductive status.
**Figure S3**. (a) The mean weighted methylation level across colonies for each genomic feature for both reproductive and sterile workers.
**Figure S4**. Graphs generated from differential expression analysis using DESEQ2 for all samples.
**Figure S5**. Graphs generated from differential expression analysis using DESEQ2 for all samples, excluding J8_24.

## References

[evl3129-bib-0001] Akalin, A. , M. Kormaksson , S. Li , F. E. Garrett‐bakelman , M. E. Figueroa , A. Melnick , et al. 2012 methylKit: A comprehensive R package for the analysis of genome‐wide DNA methylation profiles. Genome Biol. 13 10.1186/gb-2012-13-10-r87. PMC3491415 23034086

[evl3129-bib-0002] Alaux, C. , P. Jaisson , and A. Hefetz . 2006 Regulation of worker reproduction in bumblebees (*Bombus terrestris*): Workers eavesdrop on a queen signal. Behav. Ecol. Sociobiol. 60:439–446.

[evl3129-bib-0003] Amarasinghe, H. E. , C. I. Clayton , and E. B. Mallon . 2014 Methylation and worker reproduction in the bumble‐bee (*Bombus terrestris*). Proc. R. Soc. B Biol. Sci. 281 10.1098/rspb.2013.2502. PMC4027386 24523266

[evl3129-bib-0004] Amsalem, E. , O. Malka , C. Grozinger , and A. Hefetz . 2014 Exploring the role of juvenile hormone and vitellogenin in reproduction and social behavior in bumble bees. BMC Evol. Biol. 14:45. 24618396 10.1186/1471-2148-14-45 PMC4007805

[evl3129-bib-0005] Anders, S. , A. Reyes , and W. Huber . 2012 Detecting differential usage of exons from RNA‐seq data. Genome Res. 22:2008–2017. 22722343 10.1101/gr.133744.111 PMC3460195

[evl3129-bib-0006] Anders, S. , P. T. Pyl , and W. Huber . 2015 HTSeq‐A Python framework to work with high‐throughput sequencing data. Bioinformatics 31:166–169. 25260700 10.1093/bioinformatics/btu638 PMC4287950

[evl3129-bib-0007] Arsenault, S. V. , B. G. Hunt , and S. M. Rehan . 2018 The effect of maternal care on gene expression and DNA methylation in a subsocial bee. Nat. Commun. 9 10.1038/s41467-018-05903-0. PMC6110825 30150650

[evl3129-bib-0008] Ashby, R. , S. Forêt , I. Searle , and R. Maleszka . 2016 MicroRNAs in honey bee caste determination. Sci. Rep. 6 10.1038/srep18794. PMC4704047 26739502

[evl3129-bib-0009] Bao, Y. Y. , X. Qin , B. Yu , L. B. Chen , Z. C. Wang , and C. X. Zhang . 2014 Genomic insights into the serine protease gene family and expression profile analysis in the planthopper, *Nilaparvata lugens* . BMC Genomics 15 10.1186/1471-2164-15-507. PMC4085338 24952583

[evl3129-bib-0010] Bebane, P. , B. J. Hunt , M. Pegoraro , A. Jones , H. Marshall , E. Rosato , et al. 2019 The effects of the neonicotinoid imidacloprid on gene expression and DNA methylation in the buff‐tailed bumblebee *Bombus terrestris*. Proc. R. Soc. B 286:20190718. 10.1098/rspb.2019.0718 PMC6599982 31213186

[evl3129-bib-0011] Benjamini, Y. , and Y. Hochberg . 1995 Controlling the false discovery rate: A practical and powerful approach to multiple testing. J. R. Statist. Soc. 57:289–300.

[evl3129-bib-0012] Bewick, A. J. , and R. J. Schmitz . 2017 Gene body DNA methylation in plants. Curr. Opin. Plant Biol. 36:103–110. 28258985 10.1016/j.pbi.2016.12.007 PMC5413422

[evl3129-bib-0013] Bewick, A. J. , K. J. Vogel , A. J. Moore , and R. J. Schmitz . 2017 Evolution of DNA methylation across insects. Mol. Biol. Evol. 34:654–665. 28025279 10.1093/molbev/msw264 PMC5400375

[evl3129-bib-0014] Bewick, A. J. , Z. Sanchez , E. C. McKinney , A. J. Moore , P. J. Moore , and R. J. Schmitz . 2019 Dnmt1 is essential for egg production and embryo viability in the large milkweed bug, *Oncopeltus fasciatus* . Epigenetics Chromatin 12:1–14. 30616649 10.1186/s13072-018-0246-5 PMC6322253

[evl3129-bib-0015] Bloch, G . 1999 Regulation of queen‐worker conflict in bumble‐bee (*Bombus terrestris*) colonies. Proc. R. Soc. Lond. B 266:2465–2469. 10.1098/rspb.1999.0947 PMC1690486 10693816

[evl3129-bib-0016] Bonasio, R. , Q. Li , J. Lian , N. S. Mutti , L. Jin , H. Zhao , et al. 2012 Genome‐wide and caste‐specific DNA methylomes of the ants *Camponotus floridanus* and *Harpegnathos saltator* . Curr. Biol. 22:1755–1764. 22885060 10.1016/j.cub.2012.07.042 PMC3498763

[evl3129-bib-0017] Camacho, C. , G. Coulouris , V. Avagyan , N. Ma , J. Papadopoulos , K. Bealer , et al. 2009 BLAST+: Architecture and applications. BMC Bioinformatics 10:1–9. 20003500 10.1186/1471-2105-10-421 PMC2803857

[evl3129-bib-0018] Cheng, L. , and Y. Zhu . 2014 A classification approach for DNA methylation profiling with bisulfite next‐generation sequencing data. Bioinformatics 30:172–179. 24273245 10.1093/bioinformatics/btt674

[evl3129-bib-0019] Chevin, L.‐M. , R. Lande , and G. M. Mace . 2010 Adaptation, plasticity, and extinction in a changing environment: Towards a predictive theory. PLoS Biol. 8:e1000357. 20463950 10.1371/journal.pbio.1000357 PMC2864732

[evl3129-bib-0020] Collins, D. H. , I. Mohorianu , M. Beckers , V. Moulton , T. Dalmay , and A. F. Bourke . 2017 MicroRNAs associated with caste determination and differentiation in a primitively eusocial insect. Sci. Rep. 7 10.1038/srep45674. PMC5374498 28361900

[evl3129-bib-0021] Dobin, A. , T. R. Gingeras , and C. Spring . 2016 Mapping RNA‐seq Reads with STAR Alexander. Curr. Protoc. Bioinformatics 51:1–11. 10.1002/0471250953.bi1114s51 PMC4631051 26334920

[evl3129-bib-0022] Duchateau, M. J. and H. H. W. Velthuis . 1988 Development and reproductive strategies in *Bombus terrestris* colonies. Behaviour 107:186–207.

[evl3129-bib-0023] Fang, F. , E. Hodges , A. Molaro , M. Dean , G. J. Hannon , and A. D. Smith . 2012 Genomic landscape of human allele‐specific DNA methylation. Proc. Natl. Acad. Sci. 109:7332–7337. 22523239 10.1073/pnas.1201310109 PMC3358917

[evl3129-bib-0024] Feng, S. , S. J. Cokus , X. Zhang , P.‐Y. Chen , M. Bostick , M. G. Goll , et al. 2010 Conservation and divergence of methylation patterning in plants and animals. Proc. Natl. Acad. Sci. 107:8689–8694. 10.1073/pnas.1002720107 PMC2889301 20395551

[evl3129-bib-0025] Flores, K. B. , F. Wolschin , and G. V. Amdam . 2013 The role of methylation of DNA in environmental adaptation. Integr. Comp. Biol. 53:359–372. 23620251 10.1093/icb/ict019 PMC3710460

[evl3129-bib-0026] Foret, S. , R. Kucharski , Y. Pittelkow , G. A. Lockett , and R. Maleszka . 2009 Epigenetic regulation of the honey bee transcriptome: Unravelling the nature of methylated genes. BMC Genomics 10:472. 19828049 10.1186/1471-2164-10-472 PMC2768749

[evl3129-bib-0027] Foster, R. L. , A. Brunskill , D. Verdirame , and S. O'Donnell . 2004 Reproductive physiology, dominance interactions, and division of labour among bumble bee workers. Physiol. Entomol. 29:327–334.

[evl3129-bib-0028] Geva, S. , K. Hartfelder , and G. Bloch . 2005 Reproductive division of labor, dominance, and ecdysteroid levels in hemolymph and ovary of the bumble bee *Bombus terrestris*. J. Insect Physiol. 51:811–823. 10.1016/j.jinsphys.2005.03.009 15885700

[evl3129-bib-0029] Glastad, K. M. , B. G. Hunt , S. V. Yi , and M. A. Goodisman . 2011 DNA methylation in insects: On the brink of the epigenomic era. Insect Mol Biol 20:553–565. 21699596 10.1111/j.1365-2583.2011.01092.x

[evl3129-bib-0030] Glastad, K. M. , L. M. Chau , and M. A. Goodisman . 2015 Epigenetics in social insects Pp. 227–269 *in* Physiology, behavior, genomics of social insects. 1 ed. Vol. 48 Elsevier Ltd, Amsterdam, The Netherlands.

[evl3129-bib-0031] Glastad, K. M. , K. Gokhale , J. Liebig , and M. A. D. Goodisman . 2016 The caste‐ and sex‐specific DNA methylome of the termite *Zootermopsis nevadensis* . Sci. Rep. 6 10.1038/srep37110. PMC5111047 27848993

[evl3129-bib-0032] Grothendieck, G. 2017 sqldf: Manipulate R Data Frames Using SQL R package. Available at: https://cran.r-project.org/package=sqldf.

[evl3129-bib-0033] Herb, B. R. , F. Wolschin , K. D. Hansen , M. J. Aryee , B. Langmead , R. Irizarry , et al. 2012 Reversible switching between epigenetic states in honeybee behavioral subcastes. Nat. Neurosci. 15:1371–1373. 22983211 10.1038/nn.3218 PMC3518384

[evl3129-bib-0034] Hunt, B. G. , K. M. Glastad , S. V. Yi , and M. A. D. Goodisman . 2013 The function of intragenic DNA methylation: Insights from insect epigenomes. Integr. Comp. Biol. 53:319–328. 23509238 10.1093/icb/ict003

[evl3129-bib-0035] A Jones, P. 2012 Functions of DNA methylation: Islands, start sites, gene bodies and beyond. Nat. Rev. Genet. 13:484–492. 22641018 10.1038/nrg3230

[evl3129-bib-0036] Krueger, F. , and S. R. Andrews . 2011 Bismark: A flexible aligner and methylation caller for Bisulfite‐Seq applications. Bioinformatics 27:1571–1572. 21493656 10.1093/bioinformatics/btr167 PMC3102221

[evl3129-bib-0037] Krueger, F. , S. R. Andrews , and C. S. Osborne . 2011 Large scale loss of data in low‐diversity illumina sequencing libraries can be recovered by deferred cluster calling. PLoS ONE 6:4–10. 10.1371/journal.pone.0016607 PMC3030592 21305042

[evl3129-bib-0038] Kucharski, R. , J. Maleszka , S. Foret , and R. Maleszka . 2008 Nutritional control of reproductive status in honeybees via DNA methylation. Science 319:1827–1830. 18339900 10.1126/science.1153069

[evl3129-bib-0039] Langmead, B. , and S. L. Salzberg . 2012 Fast gapped‐read alignment with Bowtie 2. Nat. Methods 9:357–359. 22388286 10.1038/nmeth.1923 PMC3322381

[evl3129-bib-0040] Li, B. , L. Hou , D. Zhu , X. Xu , S. An , and X. Wang . 2018 Identification and caste‐dependent expression patterns of DNA methylation associated genes in *Bombus terrestris* . Sci. Rep. 8 10.1038/s41598-018-20831-1. PMC5799256 29402971

[evl3129-bib-0041] Li‐Byarlay, H. , Y. Li , H. Stroud , S. Feng , T. C. Newman , M. Kaneda , et al. 2013 RNA interference knockdown of DNA methyl‐transferase 3 affects gene alternative splicing in the honey bee. Proc. Natl. Acad. Sci. 110:12750–12755. 23852726 10.1073/pnas.1310735110 PMC3732956

[evl3129-bib-0042] Libbrecht, R. , P. R. Oxley , L. Keller , and D. J. C. Kronauer . 2016 Robust DNA methylation in the clonal raider ant brain. Curr. Biol. 26:1–5. 26804553 10.1016/j.cub.2015.12.040 PMC5067136

[evl3129-bib-0043] Liu, S. , A. Aageaard , J. Bechsgaard , and T. Bilde . 2019 DNA methylation patterns in the social spider, *Stegodyphus dumicola* . Genes 10:137. 10.3390/genes10020137 PMC6409797 30759892

[evl3129-bib-0044] Lonsdale, Z. , K. Lee , M. Kiriakidu , H. Amarasinghe , D. Nathanael , C. J. O'Connor , et al. 2017 Allele specific expression and methylation in the bumblebee, *Bombus terrestris* . PeerJ 5:e3798. 28929021 10.7717/peerj.3798 PMC5600721

[evl3129-bib-0045] Love, M. I. , W. Huber , and S. Anders . 2014 Moderated estimation of fold change and dispersion for RNA‐seq data with DESeq2. Genome Biol. 15:550. 25516281 10.1186/s13059-014-0550-8 PMC4302049

[evl3129-bib-0046] Lyko, F. , S. Foret , R. Kucharski , S. Wolf , C. Falckenhayn , and R. Maleszka . 2010 The honey bee epigenomes: Differential methylation of brain DNA in queens and workers. PLoS Biol. 8 10.1371/journal.pbio.1000506. PMC2970541 21072239

[evl3129-bib-0047] Martin, M . 2011 Cutadapt removes adapter sequences from high‐throughput sequencing reads. EMBnet.journal 17:10.

[evl3129-bib-0048] Matsuura, K. , N. Mizumoto , K. Kobayashi , T. Nozaki , T. Fujita , T. Yashiro , et al. 2018 A genomic imprinting model of termite caste determination: Not genetic but epigenetic inheritance influences offspring caste fate. The Am. Nat. 191:677–690. 29750562 10.1086/697238

[evl3129-bib-0049] Mcafee, A. , J. S. Pettis , D. R. Tarpy , and L. J. Foster . 2019 *Feminizer* and *doublesex* knock‐outs cause honey bees to switch sexes. PLoS Biol. 17:e3000256. 31059510 10.1371/journal.pbio.3000256 PMC6522059

[evl3129-bib-0050] Messerschmidt, D. M. , B. B. Knowles , and D. Solter . 2014 DNA methylation dynamics during epigenetic reprogramming in the germline and preimplantation embryos. Genes Dev. 28:812–828. 24736841 10.1101/gad.234294.113 PMC4003274

[evl3129-bib-0051] Mott, B. M. , J. Gadau , and K. E. Anderson . 2015 Phylogeography of *Pogonomyrmex barbatus* and *P*. rugosus harvester ants with genetic and environmental caste determination. Ecol. Evol. 5:2798–2826. 26306168 10.1002/ece3.1507 PMC4541987

[evl3129-bib-0052] Patalano, S. , A. Vlasova , C. Wyatt , P. Ewels , F. Camara , P. G. Ferreira , et al. 2015 Molecular signatures of plastic phenotypes in two eusocial insect species with simple societies. Proc. Natl. Acad. Sci. 112:13970–13975. 26483466 10.1073/pnas.1515937112 PMC4653166

[evl3129-bib-0053] Pegoraro, M. , H. Marshall , Z. N. Lonsdale , and E. B. Mallon . 2017 Do social insects support Haig's kin theory for the evolution of genomic imprinting? Epigenetics 12:725–742. 28703654 10.1080/15592294.2017.1348445 PMC5739101

[evl3129-bib-0054] Provataris, P. , K. Meusemann , O. Niehuis , S. Grath , and B. Misof . 2018 Signatures of DNA methylation across insects suggest reduced DNA methylation levels in holometabola. Genome Biol. Evol. 10:1185–1197. 29697817 10.1093/gbe/evy066 PMC5915941

[evl3129-bib-0055] Sadd, B. M. , S. M. Barribeau , G. Bloch , D. C. de Graaf , P. Dearden , C. G. Elsik , et al. 2015 The genomes of two key bumblebee species with primitive eusocial organization. Genome Biol. 16:76. 25908251 10.1186/s13059-015-0623-3 PMC4414376

[evl3129-bib-0056] Schneider, C. A. , W. S. Rasband , and K. W. Eliceiri . 2012 NIH Image to ImageJ: 25 Years of image analysis. Nat. Methods 9:671–675. 22930834 10.1038/nmeth.2089 PMC5554542

[evl3129-bib-0057] Schultz, M. D. , R. J. Schmitz , and J. R. Ecker . 2012 ‘Leveling’ the playing field for analyses of single‐base resolution DNA methylomes. Trends Genet. 28:583–585. 23131467 10.1016/j.tig.2012.10.012 PMC3523709

[evl3129-bib-0058] Shaham, R. , R. Ben‐Shlomo , U. Motro , and T. Keasar . 2016 Genome methylation patterns across castes and generations in a parasitoid wasp. Ecol. Evol. 6:7943–7953. 27878068 10.1002/ece3.2395 PMC5108247

[evl3129-bib-0059] Simola, D. F. , R. J. Graham , C. M. Brady , B. L. Enzmann , C. Desplan , A. Ray , et al. 2016 Epigenetic (re)programming of caste‐specific behavior in the ant *Camponotus floridanus* . Science 351 10.1126/science.aac6633. PMC5057185 26722000

[evl3129-bib-0060] Singer, M. , I. Kosti , L. Pachter , and Y. Mandel‐Gutfreund . 2015 A diverse epigenetic landscape at human exons with implication for expression. Nucleic Acids Res. 43:3498–3508. 25765649 10.1093/nar/gkv153 PMC4402514

[evl3129-bib-0061] Standage, D. S. , A. J. Berens , K. M. Glastad , A. J. Severin , V. P. Brendel , and A. L. Toth . 2016 Genome, transcriptome, and methylome sequencing of a primitively eusocial wasp reveal a greatly reduced DNA methylation system in a social insect. Mol. Ecol. 25:1769–1784. 26859767 10.1111/mec.13578

[evl3129-bib-0062] Supek, F. , M. Bošnjak , N. Škunca , and T. Šmuc . 2011 REVIGO summarizes and visualizes long lists of gene ontology terms. PLoS ONE 6:e21800. 21789182 10.1371/journal.pone.0021800 PMC3138752

[evl3129-bib-0063] Vojta, A. , P. Dobrinic , V. Tadic , L. Bockor , P. Korac , B. Julg , et al. 2016 Repurposing the CRISPR‐Cas9 system for targeted DNA methylation. Nucleic Acids Res. 44:5615–5628. 26969735 10.1093/nar/gkw159 PMC4937303

[evl3129-bib-0064] Wakefield, R. I. , B. O. Smith , X. Nan , A. Free , A. Soteriou , D. Uhrin , et al. 1999 The solution structure of the domain from MeCP2 that binds to methylated DNA. J. Mol. Biol. 291:1055–1065. 10518942 10.1006/jmbi.1999.3023

[evl3129-bib-0065] Wang, X. , D. Wheeler , A. Avery , A. Rago , J. H. Choi , J. K. Colbourne , et al. 2013 Function and evolution of DNA methylation in *Nasonia vitripennis* . PLoS Genet. 9:e1003872. 24130511 10.1371/journal.pgen.1003872 PMC3794928

[evl3129-bib-0066] Weiner, S. A. , D. A. Galbraith , D. C. Adams , N. Valenzuela , F. B. Noll , C. M. Grozinger , et al. 2013 A survey of DNA methylation across social insect species, life stages, and castes reveals abundant and caste‐associated methylation in a primitively social wasp. Naturwissenschaften 100:795–799. 23793297 10.1007/s00114-013-1064-z

[evl3129-bib-0067] Winkler, A. M. , M. A. Webster , D. Vidaurre , T. E. Nichols , and S. M. Smith . 2015 Multi‐level block permutation. NeuroImage 123:253–268. 26074200 10.1016/j.neuroimage.2015.05.092 PMC4644991

[evl3129-bib-0068] Woodard, S. H. , J. D. Lozier , D. Goulson , P. H. Williams , J. P. Strange , and S. Jha . 2015 Molecular tools and bumble bees: Revealing hidden details of ecology and evolution in a model system. Mol. Ecol. 24:2916–2936. 25865395 10.1111/mec.13198

[evl3129-bib-0069] Zilberman, D . 2017 An evolutionary case for functional gene body methylation in plants and animals. Genome Biol. 18:17–19. 28486944 10.1186/s13059-017-1230-2 PMC5423413

